# Aortic stiffness with bicuspid aortic valve is variable and not predicted by conventional parameters in young patients

**DOI:** 10.1186/1532-429X-17-S1-Q80

**Published:** 2015-02-03

**Authors:** Nicholas S Burris, Petter Dyverfeldt, Michael D Hope

**Affiliations:** 1 Radiology, University of California San Francisco, San Francisco, CA, USA; 2 Center for Medical Image Science and Visualization (CMIV), Linköping University, Linköping, Sweden

## Background

Bicuspid aortic valve (BAV)-related aortopathy is characterized by histologic abnormalities that result in aortic wall stiffening. Aortic stiffness has been shown to be an independent predictor of cardiovascular mortality in many settings. We sought to determine the range of aortic stiffness seen in unselected patients with BAV, and investigate associations between stiffness and various standard clinical and imaging parameters.

## Methods

BAV (n=65) and normal patients (n=10) were studied at one time point with axial conventional phase-contrast MRI through the ascending aorta. Aortic stiffness was estimated by measuring pulse wave velocity (PWV) using the flow-area (QA) method. Repeat PWV measurements were made by two independent reviewers, and average values were used for analysis. Associations between imaging and clinical/demographic parameters were investigated with Pearson's correlations. Multiple linear regression models were performed to identify independent predictors of PWV.

## Results

**Figure 1:**
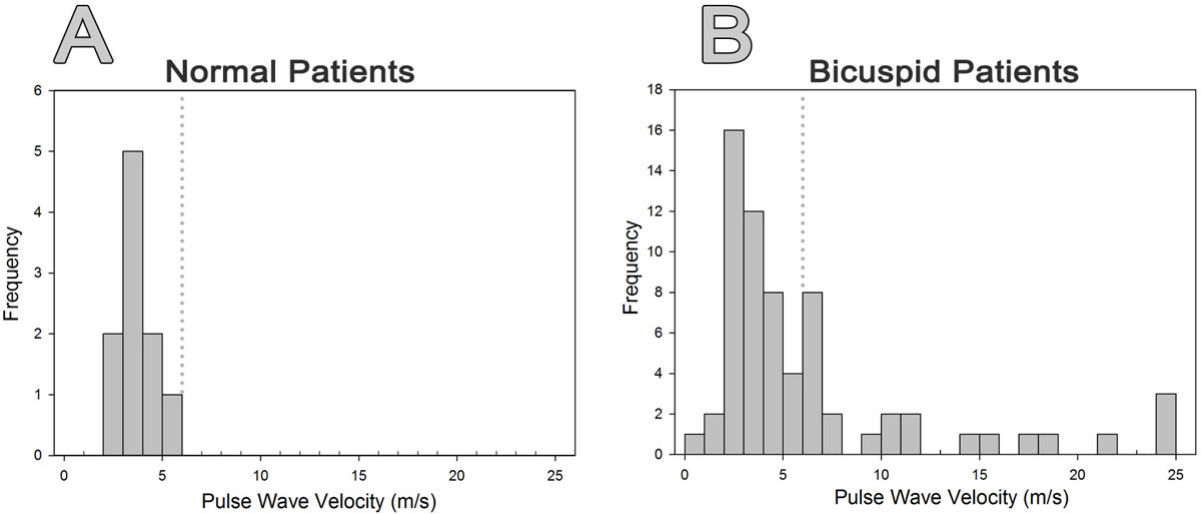
Histogram of PWV measurements. Bicuspid valve patients (A) displayed a larger range of PWV values (1.3 - 25 m/s) compared to normal patients range (B) (3.0-5.3 m/s); however, approximately two-thirds of bicuspid valve patients displayed PWV in the normal range of <6 m/s (42/65, 64%).

**Figure 2:**
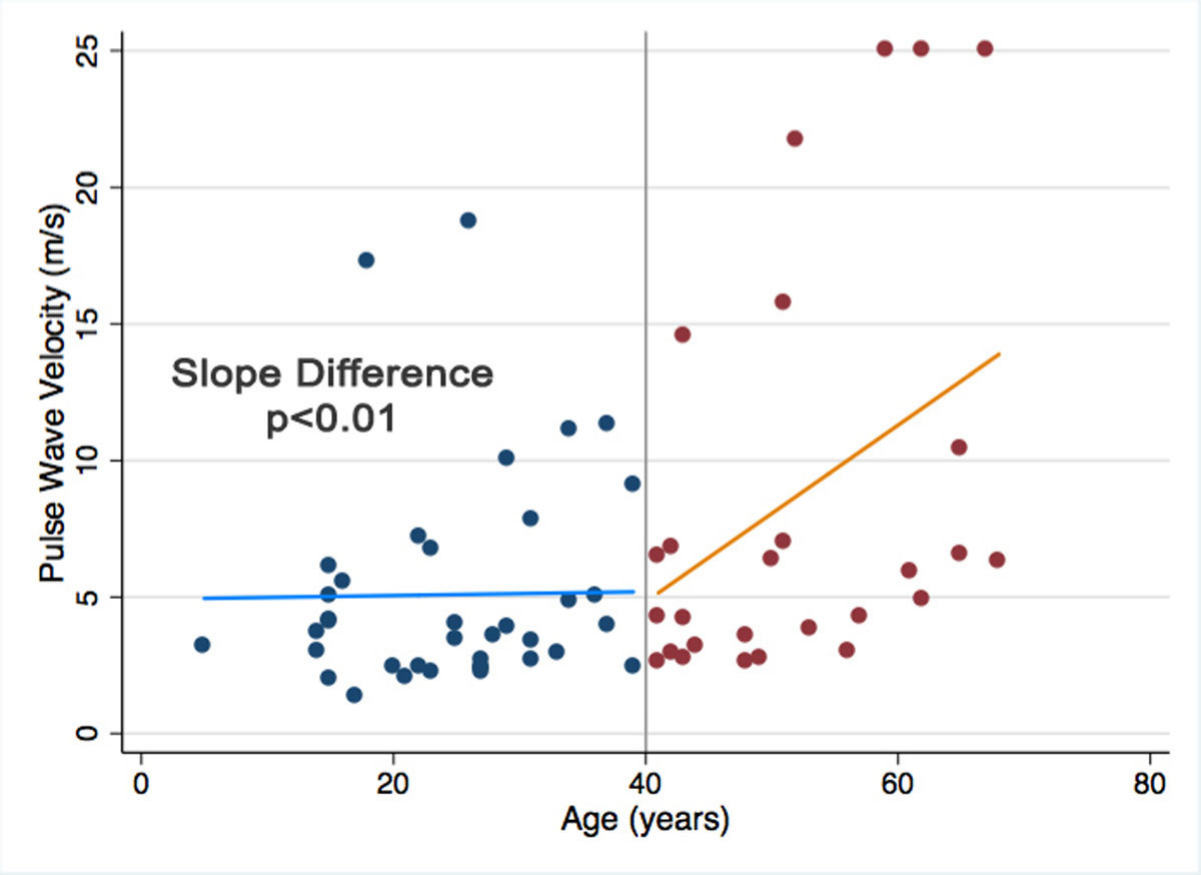
Effect of age on peak wave velocity measurements. Significantly different slopes were identified between patients <40 years old (blue dots) and those ≥40 years old (red dots) by piecewise regression with knot set at 40 years (p<0.01s).

Inter-rater reproducibility of PWV measurements was high (ICC= 0.97). There was no significant difference in age between BAV and normal patients (28.1 ± 17.2 vs. 31.2 ± 10.7 y, p=0.35). There was an overall trend toward higher PWV in patients with BAV compared to normal patients (6.53 versus 3.51 m/s, p=0.11) with a considerably higher standard deviation in BAV patients (SD of 5.88 versus 0.92 m/s)(Figure 1). No correlation was found between PWV and sex, BSA, aortic diameter, aortic valve status, leaflet fusion pattern or history of coarctation repair in BAV patients. PWV was mildly correlated with age (r=0.24, p=0.05) and history of hypertension (r=0.31, p=0.02) in the overall BAV cohort, but these correlations did not persist in a subgroup of patients <40 years old (n=37). When graphing PWV versus age, significantly different slopes were noted between the ≥40 and <40 year old subgroups by piecewise regression (p<0.01)(Figure 2). BAV patients with elevated PWV (>6 m/s, n=10) did not demonstrate significant differences in risk factors compared to those with normal PWV (<6 m/s, n=27). In the <40 year old subgroup, there were no statistically significant predictors of PWV identified by multiple linear regression.

## Conclusions

Unselected patients with BAV demonstrate a wide range of aortic stiffness compared to normal patients. Despite mild correlations with age and history of hypertension in older patients, we identified no clinical or imaging parameters that predicted aortic stiffness in younger patients, a time when many BAV patients initially present. Aortic stiffness measurements, particularly in young patients, may supplement traditional risk stratification methods for BAV patients.

## Funding

-Radiologic Society of North America Research Scholars Grant 2012-2014 (MDH).

-Swedish Research Council (PD).

-NIH T32 Training Grant 5T32EB001631-10 (NSB).

